# Mechanical failure of a curved linear array echoendoscope: loose fixation of the high frequency transducer

**DOI:** 10.1055/a-2361-4538

**Published:** 2024-07-29

**Authors:** Junichi Kaneko, Hiroki Tamakoshi, Tomoyuki Niwa, Masaki Takinami, Masafumi Nishino, Yurimi Takahashi, Takanori Yamada

**Affiliations:** 1 13773 Department of Gastroenterology, Iwata City Hospital, Iwata, Japan; 2 13773 Department of Hepatology, Iwata City Hospital, Iwata, Japan

Endoscopic ultrasound (EUS), a well-established diagnostic modality, allows for visualization of previously inaccessible anatomical regions and enables tissue acquisition and therapeutic procedures 1 2 . In interventional EUS, a curved linear array echoendoscope is first used to visualize an object with a high frequency transducer at the echoendoscope tip. A dedicated needle is then used to puncture the object under ultrasound guidance 3 . If fixation of the high frequency transducer becomes loose and rotates, it becomes difficult to visualize the puncture needle. We describe a case and in vitro studies showing the effect of mechanical failure of an echoendoscope ( Video 1 ).

A case and in vitro studies showing the effects of mechanical failure of the echoendoscope in which fixation of the high frequency transducer is loose and rotated 10° counterclockwise.Video 1

A 76-year-old man was referred to our hospital with elevated liver enzyme levels, so an EUS-guided liver biopsy was planned. A curved linear array echoendoscope (GF-UCT260; Olympus Medical Systems Corp., Tokyo, Japan) and 19-gauge Franseen needle (Acquire; Boston Scientific Corporation, Natick, Massachusetts, USA) were used. First, the left liver lobe was visualized under ultrasonographic guidance. The sheath was recognized; however, the needle was obscured ( Fig. 1 ). During puncture, the needle was visualized by moving the endoscope. Finally, liver tissue was acquired without complications. After the procedure, fixation of the high frequency transducer was found to be loose and rotated 10° counterclockwise ( Fig. 2 ). Subsequent puncture experiments were performed using tofu. The puncture needle was poorly visualized when the high frequency transducer was rotated, but it was well visualized when the rotation was restored to normal ( Fig. 3 , Fig. 4 ).

**Fig. 1:**
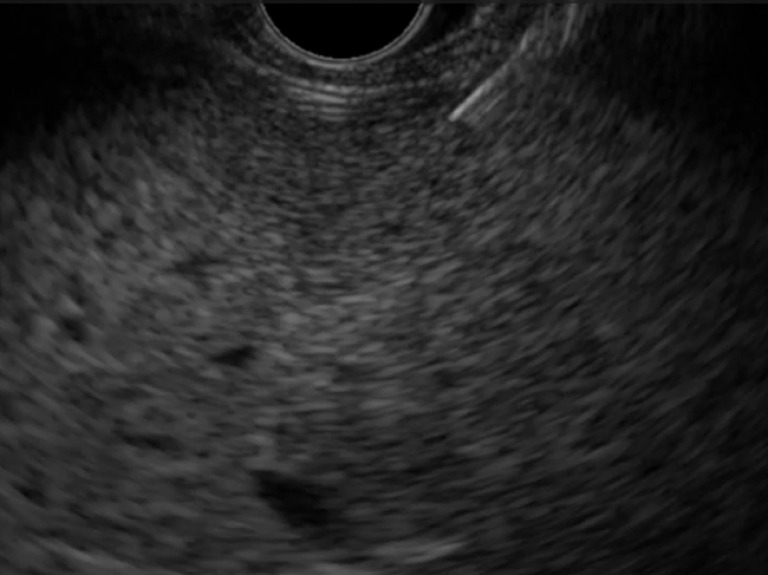
The puncture needle is not visible during endoscopic ultrasound-guided liver tissue acquisition. Subsequently, it was found that fixation of the high frequency transducer was loose and rotated 10° counterclockwise.

**Fig. 2:**
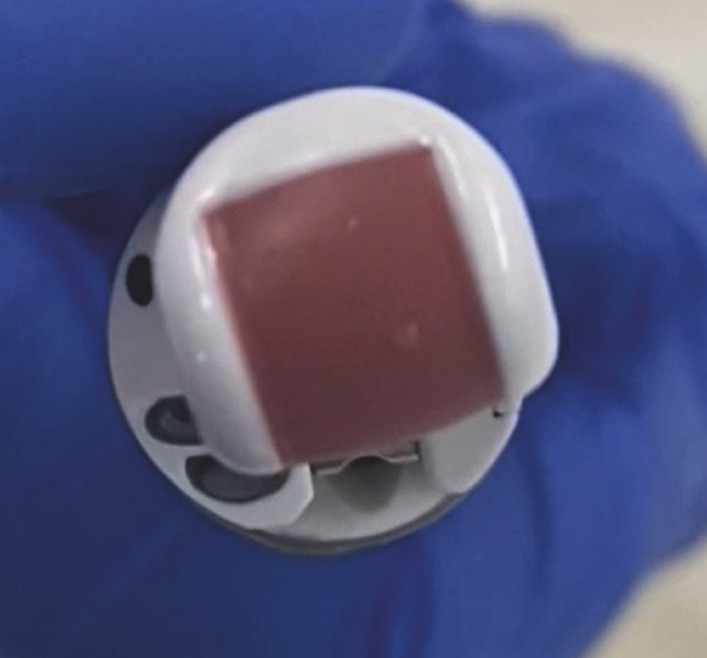
An echoendoscope with the high frequency transducer rotated 10° counterclockwise.

**Fig. 3:**
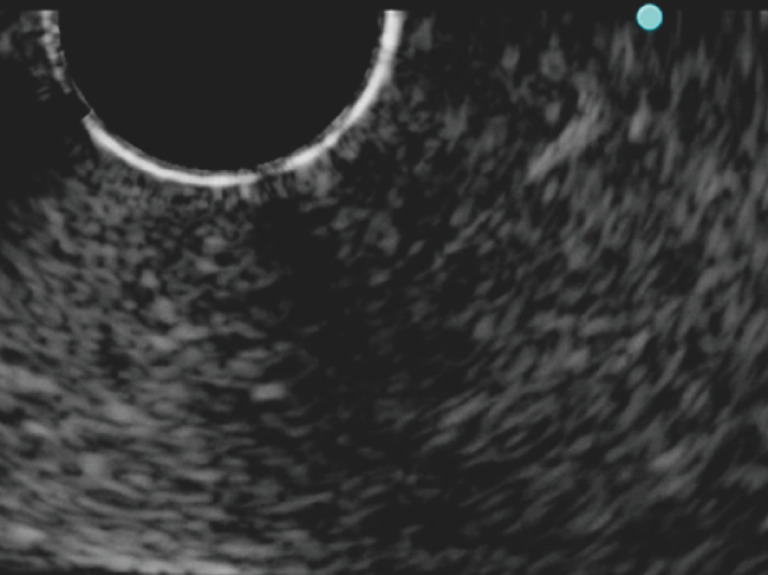
Ultrasound image showing that visibility of the puncture needle was poor when the high frequency transducer was rotated 10° counterclockwise.

**Fig. 4:**
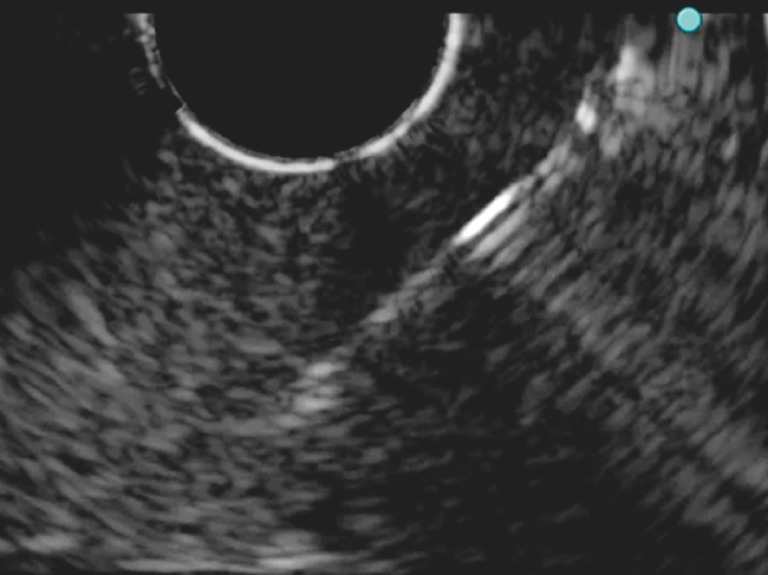
Ultrasound image showing that visibility of the puncture needle improved after correcting the position of the high frequency transducer.

The high frequency transducer was firmly fixed when this echoendoscope was purchased, and there were no instances of strong external shock waves. The cause of mechanical failure is unknown but may be due to age-related deterioration. In interventional EUS, loose fixation not only leads to an unsuccessful procedure but can also cause serious complications. Endoscopists should ensure proper fixation of the high frequency transducer before performing interventional EUS.

Endoscopy_UCTN_Code_TTT_1AS_2AF

## Notes

Endoscopy E-Videos https://eref.thieme.de/e-videos
*E-Videos* is an open access online section of the journal *Endoscopy* , reporting on interesting cases and new techniques in gastroenterological endoscopy. All papers include a high-quality video and are published with a Creative Commons CC-BY license. Endoscopy E-Videos qualify for HINARI discounts and waivers and eligibility is automatically checked during the submission process. We grant 100% waivers to articles whose corresponding authors are based in Group A countries and 50% waivers to those who are based in Group B countries as classified by Research4Life (see: https://www.research4life.org/access/eligibility/ ). This section has its own submission website at https://mc.manuscriptcentral.com/e-videos .
